# Supplementary material for: Automatic landmark annotation and dense correspondence registration for 3D human facial images
Source: BMC Bioinformatics. 2013 Jul 22;14:232. doi: 10.1186/1471-2105-14-232 (PMC3724574; doi:10.1186/1471-2105-14-232)
Supplement: Additional file 1 — Appendix. [file 1471-2105-14-232-S1.docx]

# Appendix I:

Denoting a facial mesh composed of N points by F = {p_i_} for i = 1, …, N. Suppose S is the set of M points that are within distance R around the point p_j_($1\leq j\leq N$). The best fit sphere T around p_j_ is therefore determined by two parameters, namely the center $\mathbf{O}=\left(a,b,c\right)$ and radius r. The squared distance from each point $\mathbf{p}_{k}=(x_{k},y_{k},z_{k})$, $1\leq k\leq M$in S to the surface of T is defined by

$d^{2}(\mathbf{p}_{k},\mathbf{T})=\left|(x_{k}-a)^{2}+(y_{k}-b)^{2}+(z_{k}-c)^{2}-r^{2}\right|$ (s1)

Let us denote $d^{2}(\mathbf{p}_{k},\mathbf{T})$ as $\varepsilon_{k}$, then the above equation can be expressed as

$\varepsilon_{k}=\left|(x_{k}^{2}+y_{k}^{2}+z_{k}^{2})-(2ax_{k}+2by_{k}+2cz_{k})+(a^{2}+b^{2}+c^{2}-r^{2})\right|$ (s2)

And the square distance vector is

$\mathbf{\varepsilon}=\left[\varepsilon_{1},\ldots,\varepsilon_{M}\right]^{T}$ (s3)

Our goal is to minimize the following error function

$E=\sum_{k=1}^{M} \varepsilon_{k}^{2}=\mathbf{\varepsilon}^{T}\mathbf{\varepsilon}=(\mathbf{A}-\mathbf{B}^{T}\mathbf{W})^{T}(\mathbf{A}-\mathbf{B}^{T}\mathbf{W})$ (s4)

Where

$$\mathbf{A}=\left[\begin{matrix} x_{1}^{2}+y_{1}^{2}+z_{1}^{2} \\ \vdots\\ x_{k}^{2}+y_{k}^{2}+z_{k}^{2} \end{matrix}\right]$$

This is a simple least squares problem. The solution is $\mathbf{W}=\left(\mathbf{BB}^{T}\right)^{-1}\mathbf{BA}$，and the radius is $r=\sqrt{\mathbf{W}(4)+\left(\mathbf{W}(1)^{2}+\mathbf{W}(2)^{2}+\mathbf{W}(3)^{2}\right)/4}$. The radius r is a key measurement for nose-tip recognition. In order to assess how close the point set S matches the sphere T, we introduce another measurement: the mean fitting residual, defined as $e=\sqrt{E}/M$. The smaller e is, the better S fits to a sphere.

# Appendix II:

The vector P is defined as in the main text equation 2. Denote the mean of the P vectors across the training set as$\bar{P}_{t}$, the covariance matrix is calculated as

(s5)

The eigen space U is then constructed by the eigenvectors $u_{i}$ such that

$\mathbf{C}u_{i}=\lambda_{i}u_{i}$ (s6)

where $\lambda_{i}$ is the ith largest eigen value of C. And U is given by$\mathbf{U}=\left[u_{1},u_{2},...,u_{k}\right]$. Here k is the actual number of eigen vectors to be used, which is set to 16 in our case. U therefore defines an eigen space where the sample P patches can be evaluated for similarity.

For a sample face, every point in the 2D grid is given a 21mm×21mm patch and a sample patch vector P_s_ is similarly derived following equation (2). P_s_ is then subtracted by$\bar{P}_{t}$ and projected into the eigen space U to give the weight vector

$w=\mathbf{U}^{T}(P_{s}-\bar{P}_{t})=\left[\omega_{1},\omega_{2},\cdots,\omega_{k}\right]^{T}$ (s7)

P_s_ can be reconstructed using w as. The reconstruction error can be described as

$e=(P_{s}-P_{s}')^{T}(P_{s}-P_{s}')$ (s8)

A valid landmark point should lie close to the origin point in the U space; we therefore use only points satisfying $-3\sqrt{\lambda_{i}}\leq\omega_{i}\leq3\sqrt{\lambda_{i}}$, where λ_i_ is the variance along ω_i_ across the training set. We also calculate the Mahalanobis distance from $P_{s}'$ to$\bar{P}_{t}$.

$d=\sqrt{\sum_{i=1}^{k} \frac{\omega_{i}^{2}}{\lambda_{i}}}$ (s9)

which can be another indicator of pattern similarity.
