# Supplementary figures and images for: Automatic landmark annotation and dense correspondence registration for 3D human facial images
Source: BMC Bioinformatics. 2013 Jul 22;14:232. doi: 10.1186/1471-2105-14-232 (PMC3724574; doi:10.1186/1471-2105-14-232)

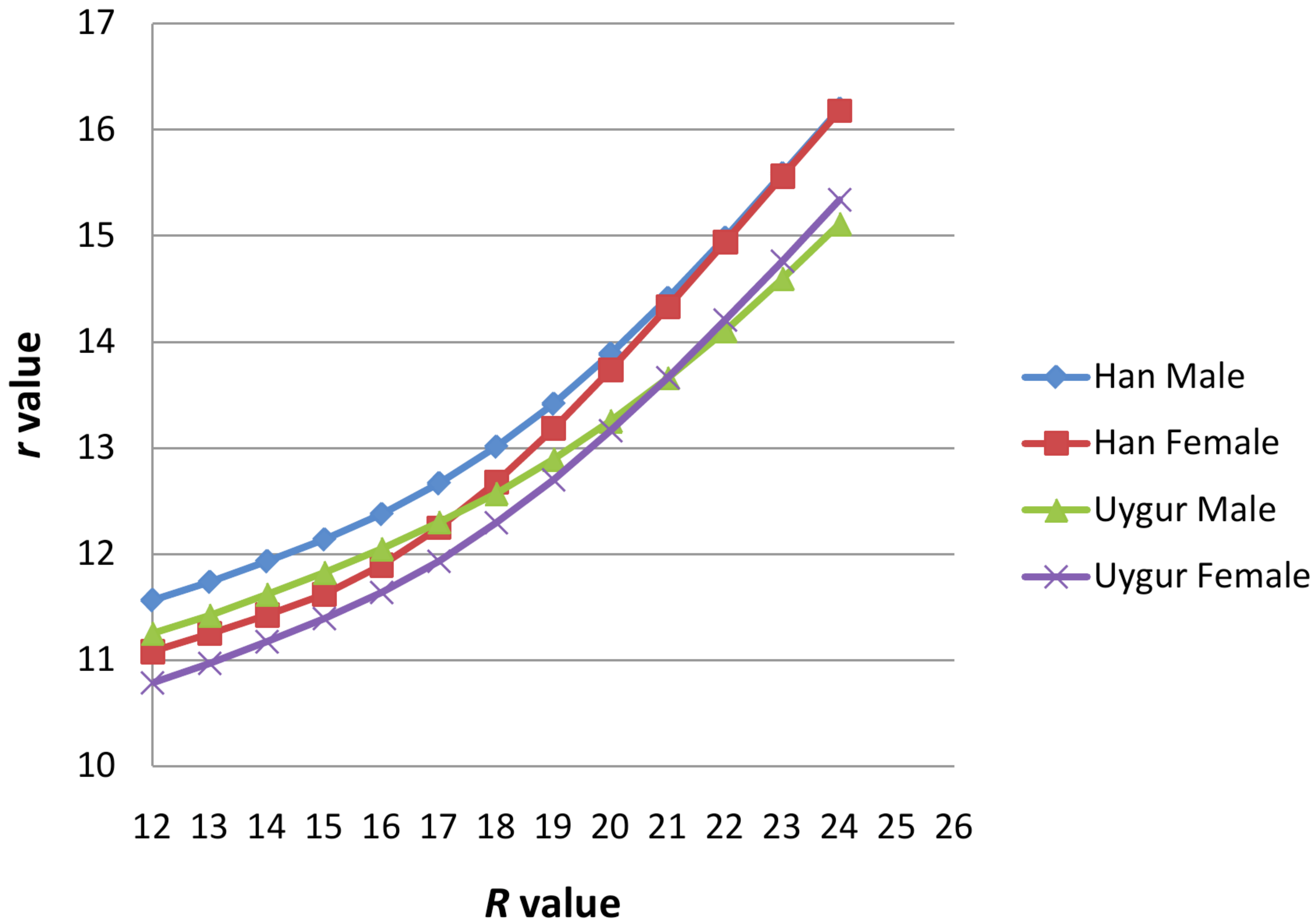

Supplement: Additional file 2: Figure S1 — Mean r value of different human groups with respect to different R values. For each group, 50 faces were analyzed. For each face, the nose tip was first annotated and all neighboring points within distance R were used to calculate the r value. [file 1471-2105-14-232-S2.pdf]

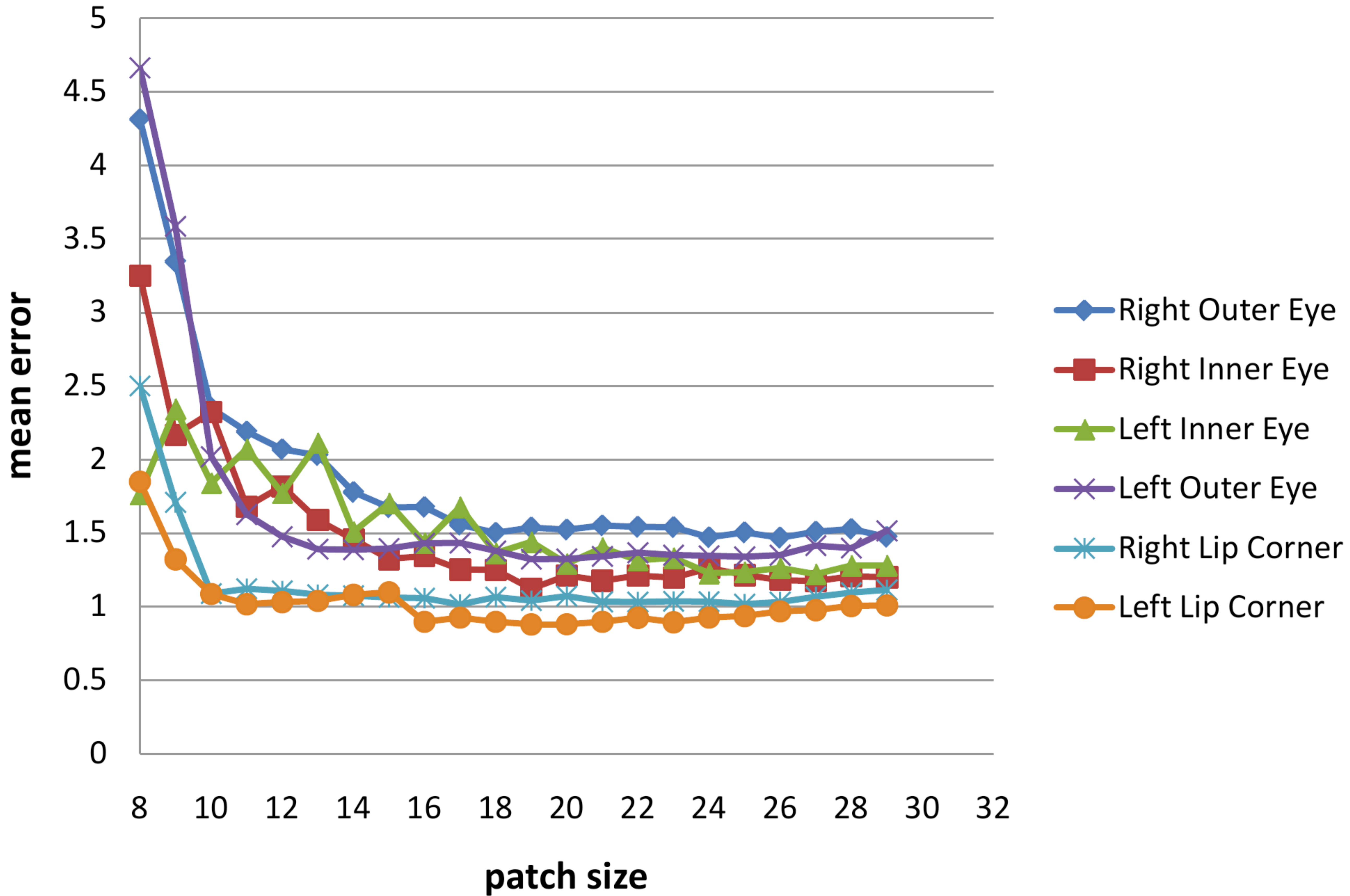

Supplement: Additional file 3: Figure S2 — Localization error (mean error) of the 6 most salient landmarks with respect to the varying patch sizes.100 faces were analyzed, of which 25 were Han male, 25 were Han female, 25 were Uygur male, and 25 were Uygur female. [file 1471-2105-14-232-S3.pdf]
